# Using Momentary Assessment and Machine Learning to Identify Barriers to Self-management in Type 1 Diabetes: Observational Study

**DOI:** 10.2196/21959

**Published:** 2022-03-03

**Authors:** Peng Zhang, Christopher Fonnesbeck, Douglas C Schmidt, Jules White, Samantha Kleinberg, Shelagh A Mulvaney

**Affiliations:** 1 Department of Computer Science School of Engineering Vanderbilt University Nashville, TN United States; 2 Data Science Institute Vanderbilt University Nashville, TN United States; 3 Department of Biostatistics Vanderbilt University Nashville, TN United States; 4 Department of Computer Science Stevens Institute of Technology Hoboken, NJ United States; 5 Department of Pediatrics Vanderbilt University Medical Center Nashville, TN United States; 6 Department of Biomedical Informatics Vanderbilt University Medical Center Nashville, TN United States; 7 School of Nursing Vanderbilt University Nashville, TN United States

**Keywords:** machine learning, type 1 diabetes, psychosocial, self-management, adolescents, behavioral medicine, ecological momentary assessment, informatics, mobile phone

## Abstract

**Background:**

For adolescents living with type 1 diabetes (T1D), completion of multiple daily self-management tasks, such as monitoring blood glucose and administering insulin, can be challenging because of psychosocial and contextual barriers. These barriers are hard to assess accurately and specifically by using traditional retrospective recall. Ecological momentary assessment (EMA) uses mobile technologies to assess the contexts, subjective experiences, and psychosocial processes that surround self-management decision-making in daily life. However, the rich data generated via EMA have not been frequently examined in T1D or integrated with machine learning analytic approaches.

**Objective:**

The goal of this study is to develop a machine learning algorithm to predict the risk of missed self-management in young adults with T1D. To achieve this goal, we train and compare a number of machine learning models through a learned filtering architecture to explore the extent to which EMA data were associated with the completion of two self-management behaviors: mealtime self-monitoring of blood glucose (SMBG) and insulin administration.

**Methods:**

We analyzed data from a randomized controlled pilot study using machine learning–based filtering architecture to investigate whether novel information related to contextual, psychosocial, and time-related factors (ie, time of day) relate to self-management. We combined EMA-collected contextual and insulin variables via the MyDay mobile app with Bluetooth blood glucose data to construct machine learning classifiers that predicted the 2 self-management behaviors of interest.

**Results:**

With 1231 day-level SMBG frequency counts for 45 participants, demographic variables and time-related variables were able to predict whether daily SMBG was below the clinical threshold of 4 times a day. Using the 1869 data points derived from app-based EMA data of 31 participants, our learned filtering architecture method was able to infer nonadherence events with high accuracy and precision. Although the recall score is low, there is high confidence that the nonadherence events identified by the model are truly nonadherent.

**Conclusions:**

Combining EMA data with machine learning methods showed promise in the relationship with risk for nonadherence. The next steps include collecting larger data sets that would more effectively power a classifier that can be deployed to infer individual behavior. Improvements in individual self-management insights, behavioral risk predictions, enhanced clinical decision-making, and just-in-time patient support in diabetes could result from this type of approach.

## Introduction

### Background

Type 1 diabetes (T1D) is a prevalent chronic illness, with increasing incidence rates reported worldwide [1,2]. It is an autoimmune disorder in which the body does not produce insulin and requires patients to perform critical self-management tasks multiple times per day [3]. Two key self-management tasks in T1D are frequent monitoring of blood glucose (BG) and administration of insulin. These tasks help manage glycemic control to avoid or delay serious short- and long-term consequences, such as retinopathy, neuropathy, and mortality [4-6]. Mealtime is a critical time for diabetes self-management.

Adolescents and young adults have the worst glycemic control of any age group [4]. For young people with diabetes, living successfully with T1D is particularly hard because of many potential psychosocial and contextual barriers to self-management [7-9]. A recommended approach to improve self-management involves promoting and supporting problem-solving skills to reduce barriers [10]. To identify problems related to self-management, patients, caregivers, and clinicians must rely on BG and insulin administration data from devices along with a patient recall of behavioral, emotional, and contextual events that could pose barriers to self-management. However, using retrospective memory or recall for events that are days or weeks in the past has been identified as generally unreliable and potentially biased in nature [11]. Unreliable recall of events in diabetes problem-solving could result in incorrect modifications to the insulin regimen.

To address the limitations of human recall and bias in health behavior research, ecological momentary assessment (EMA) methods have been developed and successfully used in a range of health conditions. In contrast to traditional assessment methods, EMA uses more frequent and in vivo ambulatory assessments of factors that affect health behaviors and decision-making. EMA methods provide a more proximal, and often more accurate, technology-mediated method to monitor and assess the contexts, subjective experiences, and processes that surround health decisions in daily life [12,13]. In particular, EMA methods provide more relevant and frequent observations per person and generate rich data to assess correlates of health behavior more accurately and identify novel correlates for intervention [14].

Many studies in the EMA literature typically use mixed effects or hierarchical linear modeling [15,16]. This analytic approach does not provide a means to automate analyses or use learning algorithms that improve and integrate incoming data over time. A promising approach for identifying such a model involves integrating EMA with techniques and tools associated with machine learning, which is a data analysis method that automates statistical model building by identifying patterns and making decisions with minimal human intervention [17,18]. Machine learning has been used with wearable sensor data and may also be useful in analyzing intensive self-report data, such as EMA. Machine learning techniques provide a viable means of examining both big and small data by providing automated classification and prediction for more feasible behavioral interventions.

### Objective

The objective of our study is to develop a machine learning algorithm to predict the risk of missed self-management. We seek to identify the momentary psychosocial and contextual factors that have an impact on T1D self-management, as assessed by EMA. To achieve these objectives, we train and compare a number of machine learning models through a learned filtering architecture (LFA) to explore the extent to which EMA data could predict the completion of two self-management behaviors: insulin administration and self-monitoring of blood glucose (SMBG). By integrating these two strategies (EMA and machine learning), we aim to provide researchers with not only a better understanding of what may hinder or promote adolescents’ adherence to their T1D regimen from a behavioral perspective but also an efficient and adaptive analytic computational method.

## Methods

### Study Design and Setting

These subanalyses analyzed data from a feasibility trial of the mobile EMA and feedback app called MyDay, which is a self-management feedback and problem-solving tool designed for adolescent patients with T1D [19]. Youth from the Vanderbilt Eskind Pediatrics Diabetes Clinic were invited to participate in a 30-day assessment period if (1) they were aged between 13 and 19 years, (2) had been diagnosed with T1D for at least 6 months, (3) owned either an Android or iPhone smartphone, (4) understood and spoke English, and (5) were willing to use a Bluetooth BG meter during the study [1]. The study was reviewed and approved by the Vanderbilt University institutional review board (IRB #150685). All parents provided consent before the adolescents provided assent. Both consent and assent were obtained before the study procedures commenced.

### Participants

A total of 48 participants were recruited for the pilot study. Of the 48 participants, 3 (6%) dropped out of the study, noting competing demands, leaving 45 (94%) for our analyses. Participants were randomized in a 2:1 ratio to the MyDay app + Bluetooth BG (meter group 31/45, 69%) and a control group (14/45, 31%). The control group provided SMBG data only using Bluetooth BG meters but did not use the MyDay app. Design processes, engagement, and momentary relationship results for MyDay have been published previously [19-21].

### Momentary Assessments and Glucose Meter Data

All SMBG data were objectively assessed using iHealth [22] glucometers. The iHealth glucometers are commercially available Bluetooth low-energy meters that can upload data automatically to the iHealth secure cloud server via their open application programming interface. Of the 45 participants, 31(69%) participants were instructed to use the MyDay app at each mealtime and bedtime to answer questions that focused on factors likely to affect diabetes self-management.

MyDay provided notifications to complete the EMA assessment personalized to typical mealtimes identified by participants. Time stamps were associated with all data entries. Only mealtime EMA was used in analyses. Variables analyzed in relation to self-management outcomes were organized into subsets. The first two domains of variables were collected for all participants: (1) *demographics* obtained at baseline (ie, gender, age, father’s education, mother’s education, family income, and race) and (2) *time variables* that were coded using the original time stamps of the collected data entries (eg, weekday, weekend, and mealtime [breakfast, lunch, and dinner]).

The next three domains of EMA data were available only for the 31 participants using the MyDay app: (3) *social context* related to who was with the youth at the time of self-management (ie, parent, sibling, alone, casual friend, close friend, other family, other person, strangers, and boyfriend or girlfriend) and where the youth was at the time of self-management (ie, home, school, work, restaurant, friends’ house, or on the road); (4) *stress, fatigue,* and *mood* levels at the reported self-management event, scored as 0 to 100, with higher scores indicating greater stress, more fatigue, and worse negative mood; and (5) selected situational *barriers* at the time of self-management event (ie, participant was rushing, feeling sick, on the road, hungry, wanting privacy, busy, without supplies, or having fun). Details of the EMA data collection process can be found in the study by Zhang et al [20].

### Outcomes

We examined three self-management behavioral outcomes:

1. Daily SMBG frequency of *<4* or *≥4* times a day; 4 glucose checks per day are generally considered as the minimum recommended [23]
2. Missed SMBG at mealtimes
3. Insulin administration at mealtimes

Data from all 45 participants were available for analyses examining the daily number of SMBG from meters. The data available for all participants were demographic and time variables. Analyses for outcomes 2 and 3 examined data from participants who used the MyDay EMA app (31/45, 69%), which obtained mealtimes.

### LFA Approach

To extract domains of variables to predict insulin administration and SMBG self-management behaviors via the training of a series of models, an LFA was created in this study as a byproduct, and a similar process was used in the study by Zhang et al [24] but not formally constructed. For this study, the LFA created and compared four machine learning models: k-nearest neighbors (KNN), logistic regression, random forest (RF), and support vector machines. These models performed binary classification for each behavioral outcome observed in this study.

KNN classifies each sample by finding its K-most similar instances in the training set and chooses the class to which most neighboring instances belong [25]. The value of k is determined by running KNN models with varying k values iteratively and selecting the k value that produces the most optimal model. Logistic regression is a statistical model that classifies a sample by predicting the probability of an output using the maximum likelihood estimation method and using a probability threshold (*P*=.50 was used in our study as the threshold such that an output with a probability of *P*≥.50 was classified as true and false otherwise) to separate the 2 classes [26]. RF is a popular ensemble learning method that trains multiple decision trees on different parts of the data set and then averages the results to improve classification accuracy [27]. The number of trees, or *estimators*, is determined by running a number of RF models with varying estimator values, such as 10, 50, and 100, and selecting the value that produced the most performant model. Support vector machines work by finding an optimal hyperplane in the feature space that optimally separates the data points into different classes [28].

**Figure 1:**
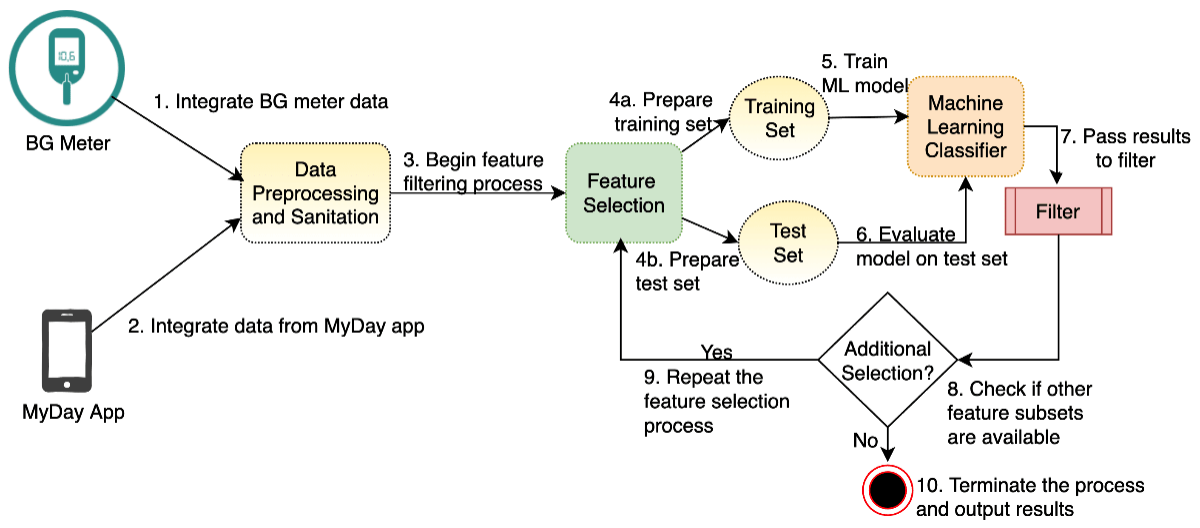
Iterative process of the learned filtering architecture. BG: blood glucose; ML: machine learning.

Figure 1 presents the workflow of this LFA and shows that the SMBG data and EMA data collected from the MyDay app were integrated as a complete data set fed into the LFA (steps 1 and 2). The LFA then performed specified data preprocessing, such as normalizing numeric values, removing entries that were empty or had many missing features, and one-hot encoding based on the type of each column (step 3). After step 3, a data filtering process began, where subsets of variables were extracted from the cleaned data either based on configurable user input, such as the names of columns that would be grouped to create a clinically meaningful, or to-be-observed, feature subset. The features were grouped as described above to create multiple data subsets. Owing to the small sample size of the data available, the data subsets were each split further for evaluating each classification model using cross-validation (steps 4a and 4b).

The LFA calculates the distribution of the target variables of each data set. If the data set is balanced, it evaluates each model using k-fold cross-validation that further splits the data into training and validation sets *k* times and produces mean values of the performance metrics. Otherwise, if the classes are unevenly distributed, it uses the stratified k-fold cross-validation to create *k* (*k*=7) splits, with each split of training and validation sets maintaining the original class distributions. The performance metrics are averaged across the results from the *k* different splits. The process is then repeated for each of the specified machine learning models (step 6).

Specifically, we used the following metrics to assess the models: (1) accuracy, which is the percentage of correct predictions; (2) precision, which is the ratio of true positives and all predicted positives that evaluates what proportion of predicted positives was actually correct; (3) recall, which is the ratio of true positives and all actual positives and calculates what proportion of actual positives was predicted correctly; (4) F1 score, which evenly weighs precision and recall; and (5) for imbalanced classification tasks, the Brier score, which is a continuous scoring loss function that evaluates the goodness of predicted probabilities in a classification task—a lower number corresponds to a stronger model and vice versa.

The classification results were then used by the filter component to compare them across all feature subsets (step 7). The filter component had a configurable tolerance value that was used to select feature subsets with relatively good classification results compared with the best-performing models. Next, the LFA checked whether additional feature groups remained to be processed (step 8). If so, feature selection was repeated to create the next data subset (step 9). Otherwise, the filtering process would terminate and output the filtered results; that is, variable groups with relatively strong predictive power of the outcomes (step 10).

The classification results were filtered to extract the best predictor groups for the target class variable. For example, if the overall performance metrics exceeded the specified threshold values (such as 15% compared with the performance metrics of the model trained with all features together), the predictor group was added to the final output queue. When all variable groups were evaluated, LFA returned the final insights obtained from the input; that is, feature groups that had significant predictive power for the outcomes observed in this study.

Although the number of observations per participant was substantial (average number of observations 60), the overall number of participants was relatively small (n=45). Thus, the collected data had some imbalance in the distribution of the outcomes, with missed mealtime insulin being a relatively less frequent event. Classification models constructed using imbalanced data sets may result in the minority class being neglected [29]. Techniques such as Synthetic Minority Oversampling Technique [30] and Tomek link [31] have been used in the literature for training imbalanced data, especially for small data sets [32-35]. However, given the small size of the population in this study, using such sampling methods would risk introducing bias and misleading results. Therefore, in this study, we used a stratified K-fold (*k*=7) cross-validation [36] evaluation method instead of random oversampling or introducing synthetic samples based on the existing data.

In stratified K-fold cross-validation, the original data set was randomly split into *k* folds. Each fold was further split into separate training and testing sets that are used to generate the evaluation metrics of a model. The distributions of the majority and minority classes within each training and testing set follow the distribution of the majority and minority classes in the original data set. After the model was trained and tested against all *k* folds, the results were averaged to represent the overall classification performance.

In addition to the machine learning methods previously described, we also used a Bayesian hierarchical regression model for the entire EMA data set that has a large number of features but a small sample size. This approach was applied to confirm the inferential power of the collected EMA data rather than focusing on which specific category was the most predictive of the outcomes.

Hierarchical modeling can capture the similarities of multiple participants within a data set while allowing estimations of individual parameters for data containing multiple participants. With the Bayesian approach, the entire data set is considered known information that is used to derive the distributions of unknown parameters of the model. It is a probabilistic model that intends to estimate the expected values or density.

In our analysis, we applied Markov chain Monte Carlo methods [37] to assist with the model formation and sampling process. Monte Carlo is a method for randomly sampling a probability distribution to approximate the desired target function. Markov chain is a sampling technique that can generate a sequence of random samples where the current sample is drawn based on the prior sample. The goal of the Markov chain Monte Carlo is to construct a Markov chain that eventually stabilizes on the desired quantity to be inferred. Specifically, we created a noncentered Bayesian hierarchical model to estimate the likelihoods of SMBG and insulin administration.

## Results

### Overview

This section first reports findings from our initial statistical analysis and then analyzes the results obtained from the LFA constructed in accordance with the methods described in the previous sections. Table 1 shows the characteristics of the sample.

**Table 1:** Characteristics of the sample (N=45).

Variable		Values
Age (years), mean (SD)		13.3 (1.7)
Female, n (%)		24 (53)
**Race or ethnicity, n (%)**		
	White	38 (84)
	African American	4 (10)
	Asian	1 (2)
	Hispanic	1 (2)
	Other	0 (0)
**Father’s education, n (%)**		
	Less than high school	1 (2)
	High school or GED^a^	13 (29)
	2-year college	7 (16)
	4-year college	15 (33)
	Graduate degree	5 (11)
	N/A^b^	4 (9)
**Mother’s education, n (%)**		
	Less than high school	0 (0)
	High school or GED	10 (22)
	2-year college	12 (27)
	4-year college	17 (38)
	Graduate degree	2 (4)
	N/A	12 (27)
**Household income (US $), n (%)**		
	<25,000	2 (4)
	25,001-35,000	3 (7)
	35,001-75,000	7 (16)
	75,001-100,000	14 (31)
	>100,000	3 (7)
	N/A	4 (9)
Duration of diabetes (years), mean (SD)		5.5 (3.7)
HbA_1c_^c^, mean (SD)		9.0 (1.9)
Use insulin pump (yes), n (%)		26 (58)

^a^GED: General Educational Development.

^b^N/A: missing values.

^c^HbA_1c_: hemoglobin A_1c_.

### Statistical Analysis

The data set was preprocessed using statistical approaches. First, it was observed that the data set contained missing values in demographic features: 9% (5/45) missing for both father’s education and household income categories and 27% (12/45) missing for mother’s education category (the percentage of missing values in each category is denoted as “N/A” entry in our report). In this study, the missing values of a feature were imputed using the mode value for features of mother’s education and father’s education and the median value for the feature of family income. Ordinal categorical variables whose order of the values were significant, such as parent education and family income level, were each transformed into a single feature with numeric values, whereas nominal variables whose significance could be assumed, such as participant race and day of the week, were converted to numeric values using one-hot encoding. Each feature was normalized using the minimum–maximum scaler such that all the final values of that feature were between 0 and 1. The source code for data preprocessing is included in Multimedia Appendix 1.

Tables 2-4 display the summary statistics of features that have *P*<.05 (ranked in ascending order) for the target feature (or dependent variable) of daily SMBG frequency, missed glucose, and insulin not administered categories, respectively. *P* value is an initial indicator that the corresponding features are statistically significant in our analysis: (1) for daily SMBG frequency, most features reported in Table 2 belong to the demographic group; (2) for SMBG, variables from the demographics, social context, barriers, and stress or mood or energy feature groups are reported in Table 3; (3) for insulin administration, variables from groups of demographics, time variables, stress or mood or energy, and barriers are reported in Table 4.

**Table 2:** Summary statistics of features with statistical significance on daily self-monitoring of blood glucose frequency.

Feature	Coefficient	SE	*P* value
Mother’s education	0.5221	0.062	<.001
Age	−0.2494	0.057	<.001
Male	0.2721	0.032	<.001
Father’s education	−0.1691	0.066	.01

**Table 3:** Summary statistics of features with statistical significance on self-monitoring of blood glucose.

Feature	Coefficient	SE	*P* value
Busy	0.1706	0.041	<.001
No supplies	0.7417	0.089	<.001
Other family	0.1436	0.038	<.001
Gender	−0.1543	0.019	<.001
Mother’s education	−0.1835	0.033	<.001
Income	−0.2569	0.039	<.001
Parent	−0.0785	0.026	<.001
Black race	−0.1064	0.038	.01
Casual	−0.084	0.031	.01
Father’s education	0.0906	0.035	.01
With sibling	0.0522	0.02	.01
In restaurant	−0.2582	0.106	.02
Hungry	−0.0436	0.021	.04
Other place	−0.2177	0.108	.045
Stress+energy	0.9274	0.466	.047

**Table 4:** Summary statistics of features with statistical significance on insulin administration.

Feature	Coefficient	SE	*P* value
Hungry	−0.0958	0.021	<.001
No supplies	0.3703	0.091	<.001
Breakfast	0.1134	0.021	<.001
Mother’s education	−0.145	0.034	<.001
Black race	−0.1637	0.039	<.001
Diabetes burnout	0.1495	0.047	<.001
Third day of week	−0.2369	0.077	<.001
Lunch	0.0695	0.022	<.001
Busy	0.1219	0.043	<.001
Second day of week	−0.216	0.077	.01
Fourth day of week	−0.2146	0.077	.01
Weekend	−0.1999	0.078	.01
Fatigue	0.0508	0.02	.01
Fifth day of week	−0.1765	0.077	.02
Low blood glucose	0.0849	0.039	.03
Gender	−0.0425	0.02	.03
Mood	−0.0919	0.043	.03
Sixth day of week	−0.1602	0.077	.04

### Daily SMBG Frequency

The average age of all participants was 13 (SD 1.7) years; 53% (24/45) were female, 84% (37/45) were White, 58% (26/45) used an insulin pump, and participants had a mean hemoglobin A_1c_ (indicating overall glycemic control) of 9.03% (SD 1.91). Additional characteristics of the sample are summarized in Table 4.

A total of 4475 BG measurements were obtained from the iHealth Bluetooth meters used by all participants (n=45). For this analysis, the demographic and time variables were studied to identify if they had any impact on the outcome of SMBG frequency per day. The measurements were aggregated on a daily basis to obtain a new data set of 1231 entries, with each entry per participant being the total number of measurements an individual had each day during the study period. SMBG frequency ranged from 1 to 12 measurements per day. If a participant did not report an entry on a particular day, the entry for that day was not assumed to have an SMBG daily frequency of 0, and hence, the entry for the participant on that day was not created.

Several distributions of daily SMBG frequencies were observed. There were 591 entries with *<4* frequency and 640 entries with ≥*4 or*. Of all the classifiers trained with the same training data, RF was the best performing model based on the overall classification metrics using the same test data. The mean and SD values of the evaluation results from the best-performing RF model are shown in Table 5 for SMBG frequencies *<4* (the source code comparing the performance of all machine learning models is included in Multimedia Appendix 1). The filter then compared the benchmark value with the outcome classification results obtained from each variable group. A tolerance value of 15% was configured for the filter to select subsets with significant predictive power. As shown in Table 5, the demographic variable group for SMBG frequency resulted in a better performance than time variables and all variables.

**Table 5:** Self-monitoring of blood glucose <4 classification results.

Feature group	Accuracy, mean (SD)	Precision, mean (SD)	Recall, mean (SD)	F1 score, mean (SD)
Demographics	75% (0.04)	75% (0.08)	72% (0.07)	74% (0.06)
Time variables	49% (0.04)	46% (0.06)	21% (0.14)	28% (0.12)
All	68% (0.03)	67% (0.06)	68% (0.06)	67% (0.03)

### Missed Mealtime SMBG and Insulin Administration

From the app group (31/45, 69%), a total of 1869 entries were associated with breakfast, lunch, or dinner and used to analyze factors that could affect SMBG and insulin administration. *Missed insulin administration* had a distribution of 1:5.72 for true (missed) versus false (administered) outcomes. In contrast, the outcome *missed SMBG* had a class distribution of 1:5.44 for true (missed) versus false (checked). LFA created classification models for each variable group (ie, demographic, time, social context, and psychosocial) using the stratified K-fold approach, as discussed previously. Similar to the previous experiment, the RF model resulted in the best classification performance in all metrics compared with other models (the source code comparing the performance of all machine learning models is included in Multimedia Appendix 2).

Tables 6 and 7 present the classification results of missed SMBG and missed insulin administration, respectively. The results showed mixed sentiments on the predictive power of individual groups of indicators on self-management behavior; however, their combined effect can be used to infer when the lack of SMBG or insulin administration occurred with high accuracy and high precision.

**Table 6:** Missing mealtime blood glucose measurement classification results.

Feature group	Accuracy (%)	Precision (%)	Recall (%)	F1 score (%)	Brier test (%)
Demographics	78	38	62	47	22
Time variables	50	13	42	20	51
Social context	61	21	55	30	25
Stress, fatigue, and mood	74	22	29	25	33
Barriers	73	33	44	33	25
All	88	78	35	48	12
All (MCMC^a^)	87	78	25	38	13

^a^MCMC: Markov chain Monte Carlo.

**Table 7:** Missing mealtime insulin administration classification results.

Feature group	Accuracy (%)	Precision (%)	Recall (%)	F1 score (%)	Brier test (%)
Demographics	65	25	65	36	36
Time variables	59	21	64	32	41
Social context	49	16	59	25	51
Stress, fatigue, and mood	74	22	28	25	32
Barriers	73	26	44	32	27
All	86	61	14	23	14
All (MCMC^a^)	85	54	15	24	15

^a^MCMC: Markov chain Monte Carlo.

## Discussion

### Principal Findings

To better understand the factors affecting the self-management behavior of adolescents with T1D, this study applied machine learning analyses to construct an LFA using demographic, BG, and momentary psychosocial and self-management data. The relative association of the 5 domains of variables for the predictability of self-management behaviors was compared using all the variables collectively as the benchmark.

For the demographic data, the results indicated that demographics were most associated with average daily SMBG frequency. These results highlight the value of social determinants of health, as defined by demographics. Although demographic factors are generally not modifiable, social determinants of health are increasingly used to adapt care to those who are most vulnerable and may not receive the full benefit of current approaches to health care [36,37].

The EMA data were able to infer nonadherence to SMBG and insulin with high accuracy and precision. Although the recall score was low, there was high confidence that the nonadherence events identified by the model are truly nonadherent. A reason for the lower recall score has to do with the small data sets that have disparities in the frequencies of observed classes or outcomes. Nonetheless, this study shows promise in the collection of larger data sets that would more effectively power a classifier that is deployable in the real world. These results also concord with our reported results from the initial statistical analysis in that (1) demographic features are correlated with daily SMBG frequencies; (2) features from each group, except for time points, have a statistically significant impact on SMBG; and (3) features from each group, except for social context, have statistically significant inferential power on insulin administration.

These results support the feasibility and value of integrating EMA and machine learning to improve behavioral assessment and automate behavioral pattern recognition in health care [18,38]. Our learned models show promise in quantifying the impact of psychosocial factors on self-management. In diabetes, stress and mood are modifiable factors that may be positively influenced by coping and problem-solving interventions [39,40]. The use of machine learning and EMA was also seen in a recent study on tinnitus (the phantom perception of sounds), where an RF classifier was applied on EMA data collected from the TrackYourTinnitus mobile app across devices to predict the mobile operating system used [41].

Social context also provided a framework for understanding risk and may be modified by interventions focused on social competence and problem solving [39]. In previous studies [42,43], behavioral observations were used to identify patterns of hand hygiene compliance monitoring, from which we obtained useful initial insights into which domains of variables had the most impact on compliance behavior.

Moving forward, the use of primarily intensive self-reported and passive psychosocial and behavioral data streams combined with machine learning could provide the basis for population-based monitoring systems to help guide automated pattern detection for clinical risk management. For example, experimental unobtrusive indicators of mealtimes are in development [44], and insulin administration is available via pumps [44]. If successful, additional passive data streams would greatly improve our methodological rigor and reach [45].

The LFA machine learning methods used here should be applied to a large, diverse sample of patients to confirm and expand the results reported in this paper. Although passive methods are increasingly used to infer behavior and psychosocial status [46,47], there are important subjective experiences, such as mood, which may continue to require self-reporting. For the foreseeable future, both self-reported real-time data and passive data, such as social networking [48], may be integrated to optimize insights for health care.

Prior research using traditional retrospective questionnaire methods has focused largely on identifying psychosocial correlates and predictors of self-management in chronic illness in general and specifically in diabetes [9]. With a few exceptions, little research using EMA has been conducted on diabetes. The few studies conducted have uniquely identified time-based factors, such as time of day and momentary negative mood, as related to self-management behaviors [49-51].

Machine learning analyses have been applied in various studies, focusing largely on the improvement of diabetes management and control. Earlier studies have constructed and fine-tuned different machine learning models to predict future BG levels based on historical physiological data [52-54], detect incorrect BG measurements [55], predict hypoglycemia [56,57], and manage insulin dosing [58] and applied it to provide lifestyle support integrating food recognition and energy expenditure [59,60]. The study results reported here advance the assessment and analysis of factors previously associated with self-management, including stress [49], mood [61,62], stigma [9,63], and social contexts [8,12]. Our study also uniquely assesses novel factors not previously studied in the T1D population, such as fatigue [64], location [65], social contexts [8], and contextual factors, such as rushing and traveling. The collected EMA data have a promising ability to infer the 2 diabetes self-management behaviors under study.

### Limitations

This study had several limitations. First, although intensive assessment resulted in a substantial number of observations per participant, the number of participants was relatively small. Although the inferential ability of this data was identified during our empirical analysis, a larger sample size in future iterations will help produce higher quality results. Second, some of the data collected here using momentary self-report, such as stress, may eventually become available as feasible passive data streams. This could reduce the burden of momentary assessment for participants and enhance the accuracy and reliability of the data. Consideration of burden should influence behavioral sampling strategies and research designs using momentary assessment. Finally, this study used a self-report of insulin administration. Moving forward, integration of insulin pumps or automated insulin administration systems will be necessary to infer insulin dosing and timing accurately.

### Conclusions

On the basis of the current findings, psychosocial context may be successfully assessed using momentary assessment, combined with physiological data, and analyzed using machine learning to optimize, and ultimately automate, health behavior insights. Similar experiments are needed with larger samples to prioritize multiple potential domains of influence on health behaviors and advance the assessment and analytic approaches used here. Future work validating self-reporting with sensor data will enhance our ability to use passive indicators of health-related behaviors. For example, experimental unobtrusive indicators of mealtimes are in development and, if successful, would greatly enhance our methodological approach [45]. The LFA machine learning methods used here will be applied to a large, diverse sample of patients to confirm and expand the results reported in this paper.

## Appendix

Multimedia Appendix 1 Source code for comparing models of daily self-monitoring of blood glucose frequency.

Multimedia Appendix 2 Source code for comparing models of self-monitoring of blood glucose and insulin administration.

## Abbreviations

BG: blood glucose
EMA: ecologic momentary assessment
KNN: k-nearest neighbors
LFA: learned filtering architectureRF: random forestSMBG: self-monitoring of blood glucose
T1D: type 1 diabetes
