# Supplementary figures and images for: Using Momentary Assessment and Machine Learning to Identify Barriers to Self-management in Type 1 Diabetes: Observational Study
Source: JMIR Mhealth Uhealth. 2022 Mar 3;10(3):e21959. doi: 10.2196/21959 (PMC8931646; doi:10.2196/21959)

### Appendix A – Source Code for Comparing Models of Daily SMBG Frequency


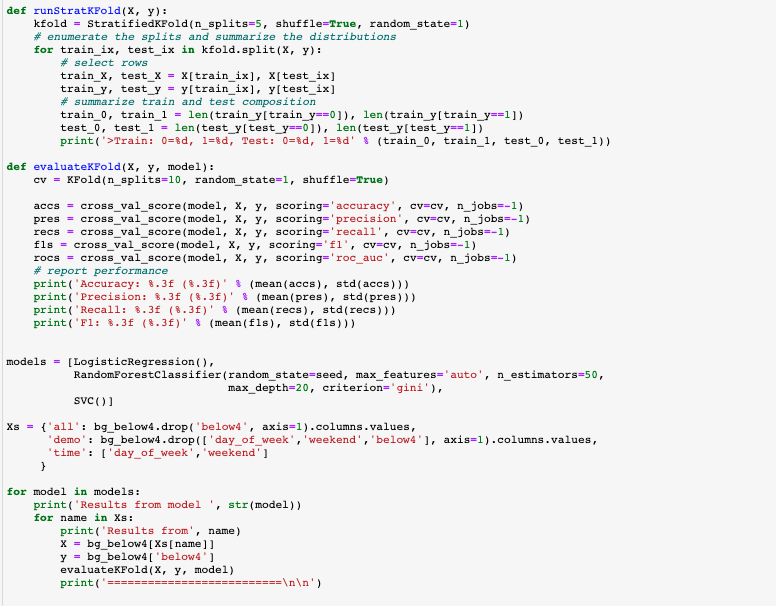

Supplement: Multimedia Appendix 1 [file mhealth_v10i3e21959_app1.docx]

### Appendix B – Source Code for Comparing Models of SMBG and IA


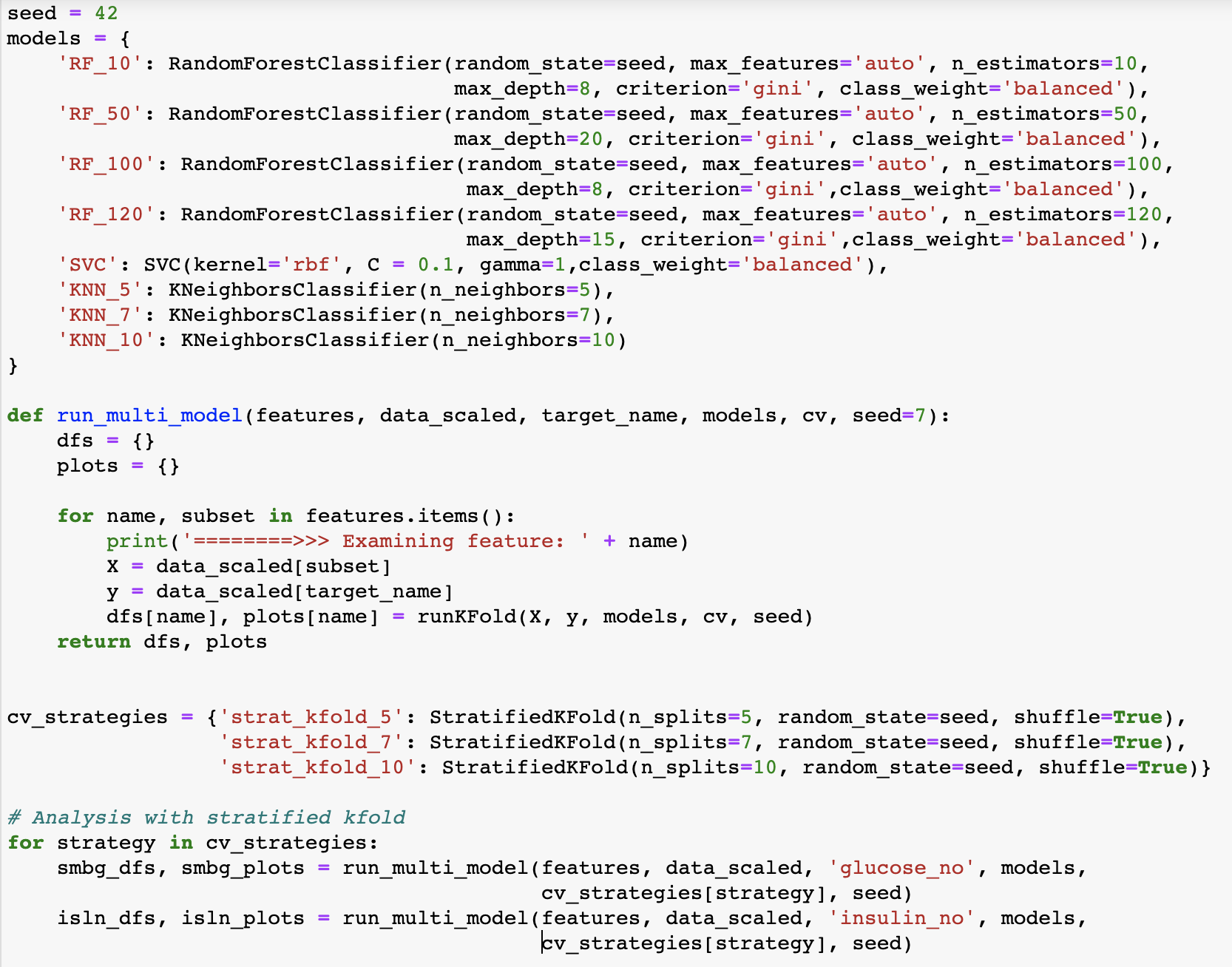

Supplement: Multimedia Appendix 2 [file mhealth_v10i3e21959_app2.docx]
